# Development of a deep inspiration breath‐hold system for radiotherapy utilizing a laser distance measurer

**DOI:** 10.1002/acm2.12011

**Published:** 2016-11-24

**Authors:** Christer Andre Jensen, Nils Skottner, Jomar Frengen, Jo‐Åsmund Lund

**Affiliations:** ^1^ Department of Oncology Ålesund Hospital Ålesund Norway; ^2^ Clinic of Oncology St. Olavs Hospital Trondheim University Hospital Trondheim Norway; ^3^ Department of Cancer Research and Molecular Medicine Norwegian University of Science and Technology Trondheim Norway

**Keywords:** breast cancer radiation therapy, DIBH, laser distance measurer, respiratory gating

## Abstract

Deep inspiration breath‐hold (DIBH) is a technique for treating left‐sided breast cancer (LSBC). In modern radiotherapy, one of the main aims is to exclude the heart from the beam aperture with an individualized beam design for LSBC. A deep inhalation will raise the chest wall while the volume of the lungs increase, this will again push the heart away from the breast to be treated. There are a few commercial DIBH systems, both invasive and noninvasive. We present an alternative noninvasive DIBH system based upon an industrial laser distance measurer. This system can be installed in a treatment room at a low cost; it is very easy to use and requires limited amount of training for the personnel and the patient. The system is capable of measuring the position of the chest wall with high frequency and precision in real time. The patient views its breathing curve through video glasses, and gets instructions during the treatment session. The system is well tolerated by test subjects due to its noninvasiveness.

## 1 Introduction

Deep inspiration breath‐hold (DIBH) during radiotherapy is a well‐established technique for treating left‐sided breast cancer (LSBC), and provides a reliable solution to lessen cardiopulmonary doses in breast irradiation.^1^, ^2^ It is also assumed that the long‐term risk of developing cardiac damage is reduced.^3^, ^4^

There are a few commercial respiratory gating systems available in the market, some are invasive and others noninvasive. Invasive instruments are not always well tolerated, and some of them use an indirect way of visualizing the motion of the chest wall. The Real‐Time Positioning Management system (Varian, Palo Alto, CA, USA) is less invasive and relies on a box with infrared markers that is placed on the patient's xiphoid process. The position of the box might vary from day to day, and its placement can influence the patient positioning and could increase the dose to the skin if placed within the field borders due to the build‐up effect.^5^ Noninvasive instruments like Sentinel and Catalyst (C‐RAD Positioning, Uppsala, Sweden) project a pattern onto the patient which is scanned with a CCD‐camera to produce a high‐resolution 3D reconstructed model of the patient.^6^, ^7^ Anzai (Anzai Medical, Tokyo, Japan) has recently released a near‐field laser sensor but no data or studies have yet been published.^8^ There have also been reports on several other systems for DIBH treatments like cine electronic imaging, infrared reflector systems, leveling marks, voluntary DIBH, breathing stick, magnets and stretching belts.^9^, ^10^, ^11^, ^12^, ^13^

In this technical note, we present a laser distance measuring system that tracks the motion of the sternum with high precision and frequency. It is possible to install the system in the clinic at a low cost, and it is very easy to use with limited training needed. The method is noninvasive, and causes no discomfort to the patient. The patient views the tracking of the sternum through video glasses. We also compared our laser system to the Catalyst surface scanning system in order to investigate if the laser system could be used as reference system for Catalyst treatments, and if the in‐house system could also be used interchangeably with the Catalyst system.

Our main aim was to develop a DIBH system and evaluate its performance. Secondly, we would like to investigate the interchangeability with the Catalyst system.

## 2 Material and methods

**Figure 1:**
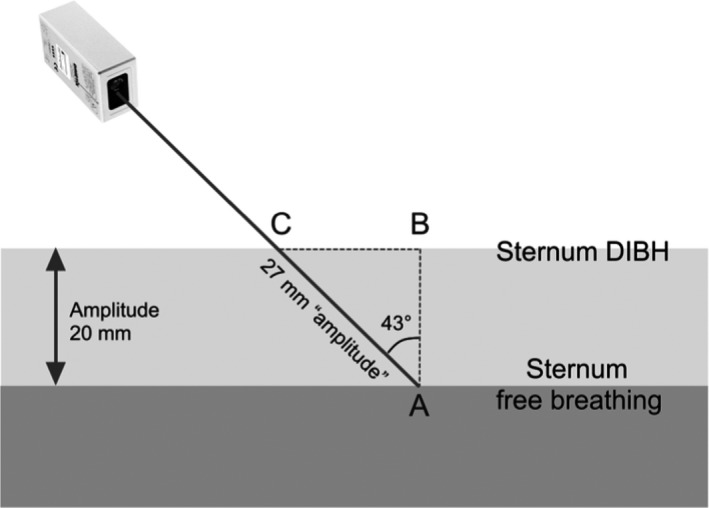
Laser pointed at point A on the patient's sternum in FB, and at point C during DIBH. Sternum amplitude of 20 mm in vertical direction, (A–B), translates to a virtual amplitude, (A–C), of 27 mm measured with the laser at 43°.

The respiratory gating system utilizes a FLS‐C10 laser distance measurer (Dimetix, Herisau, Switzerland), which is an optical distance measuring device that measures absolute distances on natural and reflecting surfaces. The laser distance sensor has an absolute accuracy of ± 1 mm @ 2σ and a repeatability of typically ± 0.3 mm @ 2σ with a measuring rate of 10 Hz in normal operation, where σ is the standard deviation. The laser distance measurer is connected to a computer via a RS‐232 serial interface and an in‐house developed program in Visual Basic (Microsoft, Redmond, USA) is used to interact with the laser, store data, and visualize the breathing curve with a small smoothing (Fig. 3a). The operator is able to adjust breathing curve baseline, amplitude, and window of the gating session, and the breathing history of the patient is visualized with an optional range. The in‐house program features audio coaching which allows the operator via buttons to deliver breathing instructions to the patient. The program is presently not capable of auto‐gating, and in order to increase patient safety and avoid mistreatments, the screen flashes in the operator's room until the first breath‐hold is performed. The laser needs to be mounted at an inclined angle in the treatment room to assure a clear view to the measuring spot on the patient's sternum. The laser system will not yield absolute amplitude in the anterior direction; this is due to the scanning displacement of the laser when the sternum is elevated from the baseline (Fig. 1). When the patient inhales, the sternum elevates, and the measuring spot moves in the inferior direction.

Catalyst is also a laser‐based scanning system mounted at an inclined angle. It has the ability to keep the measuring spot in the same superior–inferior position during breathing. As only one Catalyst system was available at our clinic, our secondary aim was to investigate if the in‐house laser system could be used at the planning CT to establish the DIBH amplitude. We would also like to examine if the laser system could be used alongside Catalyst in a treatment room; given that both systems measure a horizontal surface, there should be a fixed ratio between the vertical and inclined measurement.

### 2.A Computerized tomography

#### 2.A.1 Relative mode

**Figure 2:**
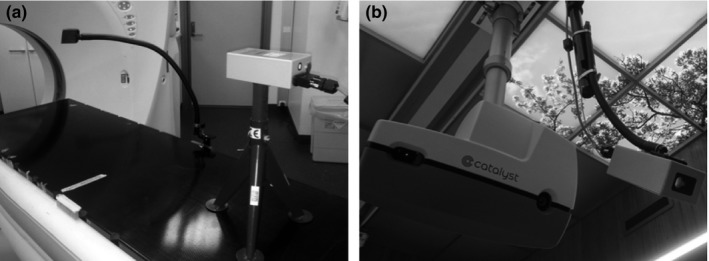
(a) Laser measurer pointing at Flexarm mirror during acquisition of planning CT. (b) Catalyst and laser measurer mounted on the ceiling in the treatment room.

Due to the inclined angle of the laser in the treatment room, a mirror jig at the CT room was developed, so that the measuring angle during CT table movement was constant and the same as during treatment. This setup ensures that the small scanning displacement along the superior–inferior axis when the patient breaths during CT and treatment will be the same. The mirror is mounted on a Flexarm (Jysk Handi, Hornslet, DK, Denmark) and can be clamped onto the side of the CT table and adjusted in the superior–inferior direction. The distance measuring laser is mounted horizontally onto a trestle and placed at the foot end of the CT table (Fig. 2a). It points toward the Flexarm mirror that deflects the laser to a 43° inclined angle, and this simple setup will follow the movement of the table during CT scanning and ensure a stable DIBH. To comply with patient safety regulations, an isolating current transformer was used to power the system.

#### 2.A.2 Absolute mode

The mirror on the Flexarm can also be adjusted to deflect the laser 90° and give a vertical readout of the sternum position. With this setup, it can be used to find the vertical reference amplitude for the Catalyst system.

### 2.B Treatment room

The laser is attached onto a Flexarm that is connected to a universal ceiling mount which, in seconds, makes it possible for the user to easily adjust the measuring spot of the laser to a suitable region regardless of the isocenter position (Fig. 2b). The laser is mounted at an inclined angle but as vertical as possible. The maximum angle will depend upon the size of the linear accelerators head; with an Elekta Versa HD this angle is 43° from the vertical plane (Fig. 1).

To validate the laser distance measurer, we performed tests against the Catalyst system. Our first test was to establish the vertical relation between the Catalyst and laser system for horizontal surfaces; this would verify the relative factor and also check for linearity. The table top was moved upward in steps while both the Catalyst and the laser system were measuring. In (Fig. 1), when the sternum is elevated, the laser will measure the distance A‐C, and not the vertical distance A‐B. This means that the laser system theoretically should measure an amplitude that is 1.37 times larger than the Catalyst for horizontal surfaces.

Seven employees were enrolled to perform a stepwise breath‐hold. The goal of the validation was to check if the two systems could be used interchangeably but with different breathing amplitudes that could be described by the ratio we found in our first test. This fixed ratio could then eventually be hard‐coded into the in‐house developed software. All test subjects used a flat WingSTEP breast board (IT‐V, Innsbruck, Austria) and knee support as fixation. Due to the inclined mounting angle of the Catalyst, it is preferable to use an angled wedge under the WingSTEP breast board in order to improve the accuracy, and we also evaluated if this would improve the results for the in‐house laser system. Measurements with an inclined breast board for four test subjects were performed.

An intra‐patient reproducibility test was also performed. This consisted of a test subject performing six independent DIBH sessions with stepwise breath‐holds while scanning with both the laser and Catalyst systems. This mimics the stand‐alone performance of the in‐house laser system when it is used both during CT and treatment.

## 3 Results

**Figure 3:**
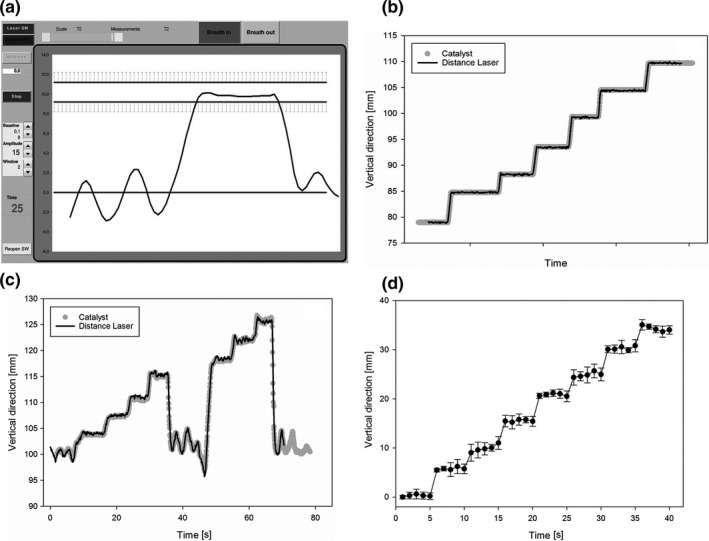
(a) Interface of the in‐house developed program that controls the laser and visualizes the breathing curve for DIBH treatment. (b) Simultaneous random vertical table position measurement results with catalyst and distance laser. (c) Plot of stepwise DIBH to max inhale for a test subjects with good correlation. The amplitude is measured in vertical direction, and the amplitude of the distance laser is divided by 1.37. (d) Reproducibility plot of six stepwise DIBH sessions for one test subject where each DIBH level is held for 5 s.

The table top was moved upward in steps while both systems were being measured (Fig. 3b). The best fit was estimated, and it was found that the amplitude of the laser system had to be divided by a factor of 1.37 with this setup. Adding a 10° wedged pillow shifted the factor to 1.14. The linearity over a 30 mm amplitude was very good, R^2^ = 1.00.

The reproducibility for one test subject was evaluated for the in‐house system. Figure 3d shows the reproducibility of six stepwise DIBHs sessions for one test subject. The mean standard deviation was 1.0 mm.

Seven employees were enrolled to see how the chest wall angle differences affected the relative differences between the two systems. It was found that the relative difference changed between the test subjects, but was in average close to the number we found with the horizontal table. At our institute, the patients have a DIBH amplitude that is 80% of the maximum inhalation, and we also investigated the best fit factor for this degree of inhalation (Table 1). We found a mean ratio of 1.51 ± 0.14 to be the best fit, ranging from 1.33 to 1.72. Figure 3c shows stepwise DIBH for one test subject, where a fixed ratio of 1.37 gave a good correlation between the two systems.

**Table 1:** Best fit ratio between laser distance measurer and Catalyst amplitude for test subjects without wedged pillow

	#1	#2	#3	#4	#5	#6	#7	Mean	Std. dev
80% Ratio	1.43	1.72	1.37	1.33	1.64	1.56	1.49	1.51	0.14

Lastly, the integration of a 10° wedge under the WingSTEP was tested, and the mean ratio for four test subjects changed from 1.57 ± 0.13 to 1.27 ± 0.09 between the in‐house laser and the Catalyst system. The standard deviation of the ratio corresponds to a ± 1.5 mm standard deviation for 20 mm DIBH amplitude when the wedged pillow is used. The maximum difference in best fit factor range was originally 0.15, but decreased to 0.12 with the wedged pillow.

## 4 Discussion

The in‐house laser system was primarily developed as a stand‐alone DIBH system; however, it was also evaluated against the Catalyst system to see if they could be used interchangeably. We found a very good correlation between the two systems for horizontal surfaces. A fixed factor of 1.37 describes the vertical ratio between the two systems for horizontal surfaces, and it showed a linear relation. Adding a 10° wedged pillow reduced this factor to 1.14. A steeper incident angle would be beneficial to both systems. A steeper incident angle would improve the reproducibility of the in‐house system and lower the standard deviation. A wedge under the WingSTEP breast board is now standard in our clinic.

We assessed the reproducibility of the laser system, and due to the fixed position of the laser in the treatment room, the reproducibility was very good over the whole DIBH range with a mean standard deviation of 1.0 mm. This reproducibility experiment mimics the performance of the system in stand‐alone mode where the measuring angle at CT is the same as in the treatment room. The laser system is currently in clinical testing as a stand‐alone system. In absolute mode, the system measures the vertical height of the sternum which will give the same amplitude as the Catalyst system, and in this mode, the laser system can be used as reference during CT for the Catalyst system.

We also evaluated the performance of our laser system against the Catalyst system in relative mode. The correlation was good in the internal study with seven test subjects, but the test subjects had variations in sternum angle that led to differences in the ratio between the two systems. At the 80% maximum inhale level, a mean ratio of 1.51 was the best fit between the two systems, which is somewhat larger than the 1.37 ratio found for horizontal surfaces. A difference of 0.14 would imply that the mean sternum angle of the seven test subjects was inclined in the superior direction from the horizontal plane. When the lungs are filled with air, the xiphoid process will elevate while the cranial parts of the chest wall will remain relatively stable, and hence this will lead to a small angle on the chest wall even if the gating spot was chosen to be at a horizontal part of the sternum. This would imply that an angled wedge under the WingSTEP breast board could be a good compromise, and shift the mean ratio closer to 1.00. The test with the angled wedge under the WingSTEP shifted the mean ratio from 1.57 ± 0.13 to 1.27 ± 0.09 between the laser and the Catalyst systems, and this will improve the interchangeability of the two systems.

When the same inclined angle is used both during CT and treatment, the scanning displacement of the laser system will not influence the treatment results in relative mode provided that both the positioning and breathing performance is kept similar. This is the case if the laser system is used both for reference at CT and during treatment sessions. If the Catalyst and laser system are used interchangeably with the mean patient ratio, there could be a small error in the amplitude in the range of 0–3 mm if one does not take the angle of the sternum into account. The maximum difference in ratio was 0.12 with the angled pillow, which for 20 mm amplitude translates to a 2.4 mm error. A small error would have little impact on the quality of the treatment if the patient is repositioned after portal imaging, but it will influence the amount of air inhaled and hence the cardiopulmonary dose might be different than planned. If the two systems are used interchangeably, a wedged pillow is recommended.

Anzai Medical has recently released a resembling commercial solution but there is no published data in the literature to date.^8^ Their system will have to be mounted each day after the patient is aligned to localization lasers, increasing setup time. This will also be susceptible to setup variations and possibly also varying inclination angle. Due to the close proximity to the sternum, it might also end up in the radiation field.

We present a deep inspiration breath‐hold system for planning and treating breast cancer patients. Our laser system is capable of measuring the position of the chest wall with high frequency and precision in real time. The tests we performed showed good reproducibility and the system can be used in absolute or relative mode. In absolute mode, it can be used as reference for Catalyst at CT, and in relative mode, it can be used as stand‐alone system with high reproducibility. The system is currently in clinical testing as a stand‐alone system in relative mode. The most important features of our in‐house system are its simplicity, its noninvasiveness, and its low cost for performing DIBH.
